# US Coverage Changes During Medicaid Unwinding in 2023

**DOI:** 10.1001/jamahealthforum.2025.3887

**Published:** 2025-10-10

**Authors:** Adrianna McIntyre, Molly Morein, Jinwoo Kim, Jose F. Figueroa, Benjamin D. Sommers

**Affiliations:** 1 Department of Health Policy and Management, Harvard T.H. Chan School of Public Health, Boston, Massachusetts; 2 Department of Medicine, Brigham & Women’s Hospital, Harvard Medical School, Boston, Massachusetts

## Abstract

**Importance:**

The unwinding of Medicaid’s continuous coverage provision, beginning in April 2023, disenrolled more than 25 million people. Effects on overall insurance coverage—including reenrollment in Medicaid—and affordability of care remain unclear.

**Objective:**

To estimate variation in coverage losses during unwinding of the Medicaid continuous coverage provision by state and demographic groups and evaluate trends in new Medicaid applications before, during, and after the COVID-19 public health emergency.

**Design, Setting, and Participants:**

In this cross-sectional study, using administrative data from the US Centers for Medicare & Medicaid Services (CMS), state-level Medicaid terminations as a share of pre-unwinding Medicaid enrollment were estimated during the first year of unwinding and new application rates were examined. CMS administrative enrollment data were compared with quarterly estimates from the National Health Insurance Survey (2019-2023), examining demographic changes in self-reported Medicaid, uninsurance, and cost-related barriers to care among low-income individuals in the second half of 2023 using an interrupted time series. A nationally representative sample of US residents younger than 65 years applying to and enrolled in Medicaid in federal survey data was included. Subgroup analyses were conducted by health status, race and ethnicity, education, pregnancy status, and age. Survey data were collected from January 2019 to December 2023, administrative data were collected from January 2019 to April 2024, and data were analyzed from November 2024 to July 2025.

**Exposures:**

The unwinding of the Medicaid continuous coverage provision, under which people could maintain program enrollment even if their eligibility status changed.

**Main Outcomes and Measures:**

Enrollment in Medicaid (administratively reported and self-reported), percentage uninsured, cost-related delays in care, and new applications to Medicaid in states with high and low procedural termination rates.

**Results:**

As a share of pre-unwinding Medicaid enrollment, the median (range) state termination rate during unwinding in 2023 was 26.6% (8.3-55.3). Net enrollment decreases, including new and returning enrollees, were smaller (median [range] decrease, 13.9% [−15.0 to 30.2]). New applications fell 29.8% (from a mean [SD] of 27 914.20 [3669.97] to 19 589.61 [2622.08]) during the COVID-19 pandemic but increased 29.0% (from a mean [SD] of 19 589.61 [2622.08] to 25 273.11 [3650.18]) during unwinding. Gaps between administratively measured and survey-measured enrollment grew during the COVID-19 pandemic but narrowed during unwinding. During unwinding, survey-reported Medicaid enrollment decreased among women (−2.74 percentage points; 95% CI, −4.82 to −0.66), healthier individuals (−2.65 percentage points; 95% CI, −4.84 to −0.46), young adults (−3.37 percentage points; 95% CI, −6.32 to −0.42), recently pregnant adults (−8.19 percentage points; 95% CI, −16.35 to −0.03), and White individuals (−2.85 percentage points; 95% CI, −5.36 to −0.34). Changes in uninsurance overall were modest but rose significantly among healthier individuals. Cost-related barriers to care rose among White individuals, college-educated individuals, and healthier individuals.

**Conclusions and Relevance:**

In this cross-sectional study, millions lost Medicaid during unwinding (with wide state variation), but survey data indicated smaller coverage reductions. Re-enrollment after termination and unawareness of COVID-19 pandemic coverage partially explain this discrepancy. Coverage losses were concentrated among certain demographic groups, with some evidence of reduced care affordability. These results may inform expectations about recent Medicaid proposals that would increase administrative barriers to coverage.

## Key Points

**Question:**

How did coverage losses during unwinding of the Medicaid continuous coverage provision vary across different measures, states, and demographic groups?

**Findings:**

In this cross-sectional study, net Medicaid enrollment loss during unwinding varied substantially across states and was lower than the sum of terminations during this time. Coverage losses were concentrated among several demographic groups, with healthier individuals more likely to become uninsured and report barriers accessing care.

**Meaning:**

Discrepancies between disenrollments and net losses reflect both churn and coverage transitions; state variation observed during unwinding may inform expectations about pending Medicaid reforms.

## Teaser

This cross-sectional study estimates variation in coverage losses during unwinding of the Medicaid continuous coverage provision by state and demographic groups and evaluates trends in new Medicaid applications before, during, and after the COVID-19 public health emergency.

## Introduction

Over the course of about 18 months, starting in early 2023, more than 25 million people—nearly 8% of all US residents—had their Medicaid coverage terminated.^6^ This precipitous decline, arguably the largest drop in Medicaid enrollment in the program’s history, followed on the heels of equally unprecedented enrollment growth during the COVID-19 pandemic. However, despite concerns of policymakers and other key stakeholders, this mass Medicaid exodus has not been reflected in federal estimates of the uninsured rate.

These marked enrollment fluctuations between 2020 and 2024 were driven by Medicaid policy changes related to the COVID-19 pandemic and resulting public health emergency (PHE). In March 2020, Congress implemented a continuous coverage provision in Medicaid, under which states were offered additional federal funding in exchange for prohibition against removing beneficiaries from Medicaid due to eligibility changes.^1,2^ This provision remained in effect through March 2023, after which states were permitted to resume eligibility redeterminations for Medicaid enrollees—ordinarily an annual process—thus unwinding continuous coverage.

Estimates summing Medicaid terminations during unwinding overstate total coverage losses because they do not account for people who transitioned to other insurance or who later returned to Medicaid (so-called churn).^3,4^ Medicaid churn was a particular concern because procedural terminations—terminations where the state could not ascertain an individual’s eligibility status, often because they did not receive or could not complete renewal paperwork—represented most terminations in most states.^5^ A 4-state survey from late 2023 found that nearly one-half of low-income adults leaving Medicaid became uninsured; other studies have found that Medicaid disenrollment was higher among those with mental health conditions, with mixed findings on racial and ethnic disparities in coverage loss.^6,7,8,9^ Research also suggests that many people who remained in Medicaid during the COVID-19 pandemic did not recognize that their coverage had continued, describing themselves as uninsured or as having other insurance, even though Medicaid should have remained in place for nearly all individuals.^10,11,12^

Surfacing dynamics that explain the disconnect between administrative and survey-reported Medicaid enrollment is critical to understanding the broader effects of unwinding on uninsured rates and health care affordability. Furthermore, characterizing which states and populations were most affected by these policy changes and how beneficiaries perceived their coverage status can inform expectations about recently implemented policies extending 12-month continuous coverage for postpartum and child Medicaid enrollees, as well as federal proposals to substantially reform Medicaid eligibility processes.^13,14^ Finally, these results may also inform expectations as policymakers contemplate changes to Medicaid that would make eligibility redeterminations more frequent and otherwise increase administrative barriers in the program, such as work requirements.^15^

Many preliminary estimates of the effect of unwinding on coverage outcomes relied on the US Census Bureau Household Pulse Survey, which offered timely estimates during and following the COVID-19 pandemic but also had major limitations and has since been discontinued.^9,10,16^ The Household Pulse Survey had a markedly lower response rate compared with other federal surveys capturing health insurance coverage (approximately 6% vs 49% in the 2023 National Health Interview Survey [NHIS], for example),^17,18^ and response rates were particularly low for lower-income individuals, increasing concerns of nonresponse bias. The survey also did not collect information on children’s insurance status, providing an incomplete picture of the effects of unwinding.^17^

In this article, we offer an overarching picture of Medicaid coverage before, during, and after the COVID-19 PHE and evidence for 3 phenomena at the root of the disconnect between survey and administrative measures of Medicaid enrollment: enrollee confusion about their coverage status, churn back into the Medicaid program, and transitions to new, non-Medicaid sources of coverage.

## Methods

### Data Sources

We rely on several sources of administrative and survey data. We used CMS monthly enrollment reports (from January 2019 through April 2024), which measure state-level administrative enrollment in Medicaid and Children’s Health Insurance Program, separately for children and adults, and new applications to the program (not stratified by age).^19,20^ To convert administrative enrollment counts into population percentages, we constructed denominators using the American Community Survey. In addition to the enrollment reports, we used administrative CMS data on state-level eligibility redetermination outcomes, spanning March 2023 through April 2024. Quarterly estimates of self-reported coverage for children and adults and health care affordability outcomes were measured using the NHIS public data files.^21^ Consistent with 45 CFR §46, this study using deidentified, publicly available survey and administrative data did not require institutional review board approval or informed consent. This study followed Strengthening the Reporting of Observational Studies in Epidemiology (STROBE) reporting guideline for a cross-sectional study.

### Statistical Analysis

To evaluate trends in the Medicaid undercount—the gap between administratively measured enrollment and survey-reported enrollment, which may partially reflect enrollees’ confusion about their coverage status—before, during, and after the PHE, we compared self-reported Medicaid enrollment in the 2019-2023 NHIS to the share of the total population enrolled in Medicaid per CMS enrollment data. This quarterly (ie, 3-month means) analysis using the NHIS adult and child files permits evaluation of trends that may be obscured in annual surveys like the American Community Survey. NHIS estimates were survey-weighted to be representative of the full national population. We converted administrative enrollment to percentages by estimating year-specific and age-specific (adult vs child) denominators using the American Community Survey.

To better understand the potential role of churn, we compared cumulative state-level Medicaid terminations to net changes in Medicaid enrollment over the first 12 months of unwinding, benchmarking losses against March 2023 enrollment (the last month of continuous coverage).

We evaluated application trends before, during, and after the PHE. Specifically, we measured the mean number of new Medicaid applications each month, estimating relative changes in average application counts before the continuous coverage provision was in effect (January 2019 through February 2020), during continuous coverage (May 2020 through March 2023), and during unwinding (April 2023 through April 2024). We omitted March to April 2020 from this comparison due to the brief spike in applications accompanying the onset of the COVID-19 pandemic. We estimated application trends nationally and stratifying states by whether their average procedural termination rate was above or below the national median. We analyzed mean application counts and repeated this analysis standardizing application rates as a share of total state Medicaid enrollment in each month to account for variation in the size of state Medicaid programs. We also estimated bivariate associations between terminations and new applications (as a share of monthly Medicaid enrollment) during unwinding. Application counts were lagged 4 months, because people may reenter Medicaid without a new application during a 3-month reconsideration window.^22^

Analyses of application trends included data from 29 states, due to the omission of states with missing or incomplete data. Our overall unwinding and survey-based estimates included all 50 states and Washington, DC. See eTable 1 in Supplement 1 for additional details.

Finally, to examine whether coverage dynamics differentially affected particular subgroups, we used an interrupted time series model and NHIS data to examine changes in survey-reported health insurance coverage and cost-related barriers to care (defined as not receiving any care or delaying care in the past 12 months due to costs) before and after the PHE and unwinding periods. Subgroup analyses were conducted based on self-reported race (American Indian or Alaska Native, Asian, Black/African American, White, or any other group) and ethnicity (Hispanic or non-Hispanic), education level, age, urban vs rural residence, sex, self-rated health, and currently pregnant and/or giving birth within the past year. We restricted these analyses to individuals aged 0 to 64 years with incomes at or below 200% the federal poverty level, the population presumably most vulnerable to coverage disruptions during unwinding.

Data spanned January 2019 to December 2023. We modeled 3 discrete time periods based on quarterly time trends, allowing each to have its own slope: before PHE (20 quarters), the period of the PHE continuous coverage provision (starting in the second quarter of 2020; 16 quarters), and the unwinding period (starting in the third quarter of 2023; 3 quarters). The third quarter of 2023, rather than the second, was selected because overall Medicaid enrollment in CMS data did not begin to decline until that point; it was not until the summer of 2023 that all states were actively engaged in the unwinding process.

All models used survey-weighted linear regressions. For each regression model, we reported coefficients with 95% CIs. Statistical significance was set at *P* < .05, using 2-tailed tests. All analyses were conducted using Stata/SE version 18.5 (StataCorp). Additional details on the regression approach and subgroup definitions are available in the eMethods in Supplement 1.

## Results

### Administrative vs Self-Reported Medicaid Enrollment

**Figure 1.**
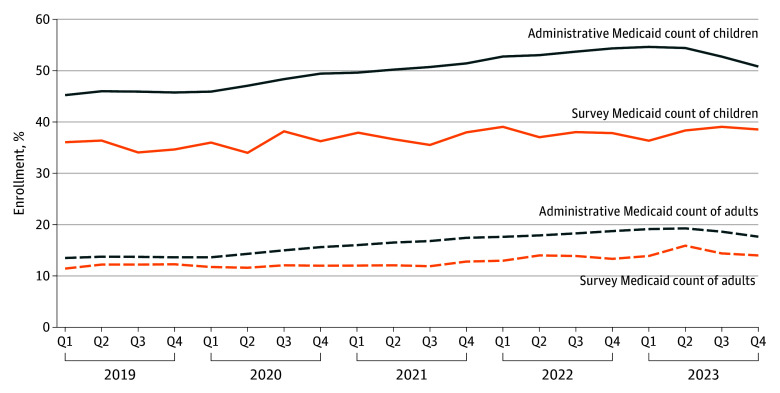
Quarterly Enrollment in Medicaid Based on Administrative Data (US Centers for Medicare & Medicaid Services) vs Survey Data (National Health Interview Survey) for Adults vs Children, 2019-2023 Administrative enrollment data are from Centers for Medicare & Medicaid Services monthly enrollment reports. To convert administrative enrollment counts into population percentages, we used the American Community Survey to estimate state-specific, year-specific, and age-specific population sizes. We report quarterly enrollment, with quarter 1 (Q1) including data from January, February, and March; Q2, April, May, and June; Q3, July, August, and September; and Q4, October, November, and December. Survey estimates measured self-reported Medicaid enrollment in the 2019-2023 National Health Interview Survey adult and child files. These estimates were survey-weighted to be representative of the national population. Quarterly National Health Interview Survey estimates used all US residents to produce comparable results between the survey estimates and Centers for Medicare & Medicaid Services administrative data.

During the study period, total Medicaid enrollment, measured administratively, ranged from 72.0 million to 94.4 million, depending on the quarter. Figure 1 plots administrative and survey estimates of Medicaid enrollment as a share of the US population from 2019 through 2023. Medicaid coverage grew steadily while the continuous coverage provision was in effect. However, the Medicaid undercount grew wider during this time and was worse for children, peaking at a difference of 18.4 percentage points (54.6% vs 36.2%) for children compared with 5.2 percentage points (19.1% vs 13.8%) for adults in the first quarter of 2023, just before unwinding commenced. These gaps began to narrow during unwinding.

### Terminations, Net Enrollment Changes, and Applications to Medicaid

**Figure 2.**
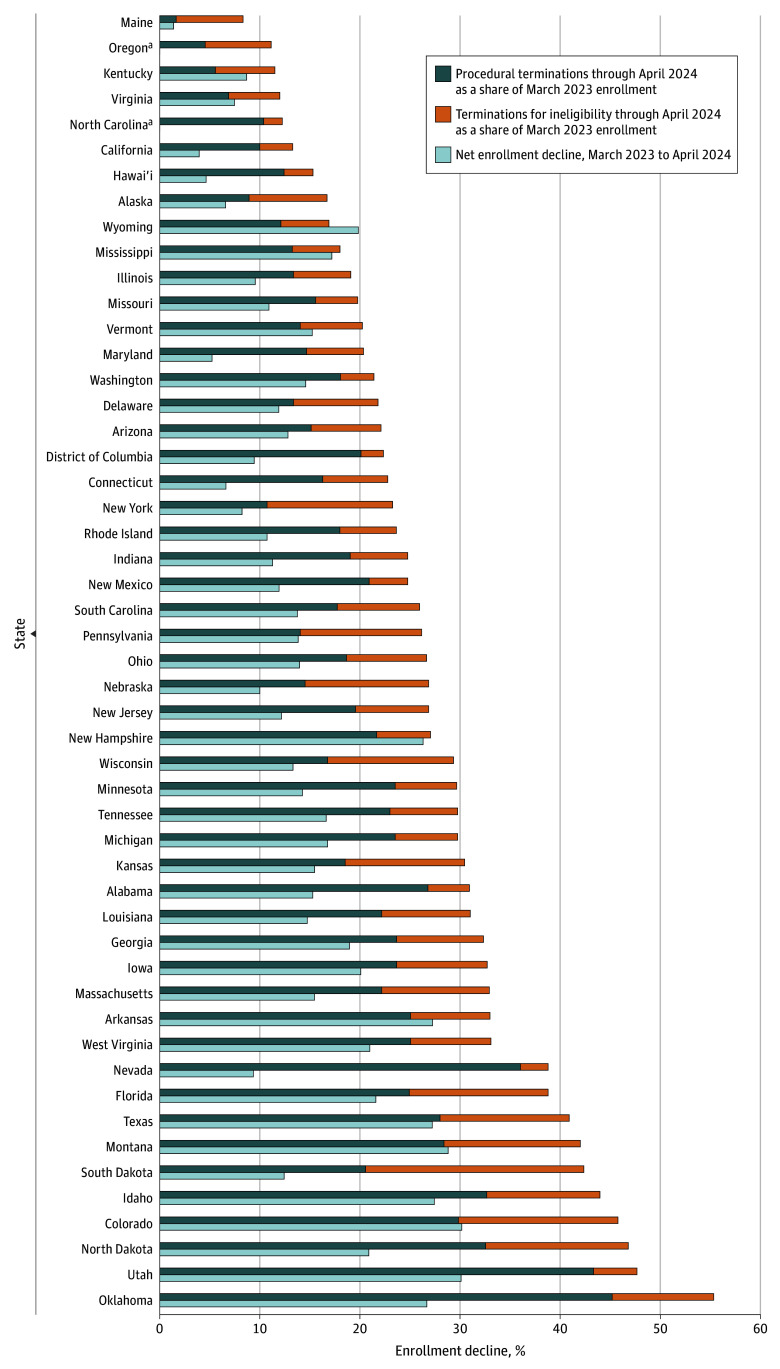
Cumulative Terminations vs Net Decreases in Medicaid Enrollment, March 2023 to April 2024 Data are from US Centers for Medicare & Medicaid Services Medicaid enrollment reports. Cumulative terminations were estimated using April 2024 Consolidated Appropriations Act report data and compared with Medicaid enrollment in March 2023, when the continuous coverage provision ended. Procedural terminations refer to terminations where the state could not definitively ascertain an individual’s eligibility status. March 2023 enrollment data are drawn from monthly Centers for Medicare & Medicaid Services enrollment reports. Monthly enrollment reports were also used to estimate state-level changes in net Medicaid enrollment from March 2023 through April 2024. ^a^We do not report net enrollment changes for North Carolina and Oregon because net enrollment increased rather than decreased in these states. North Carolina expanded Medicaid under the Affordable Care Act, effective December 1, 2023; enrollment increased 15.0% over the time period of interest (March 2023 to April 2024). Oregon did not start conducting procedural terminations until October 2023, months after most states had begun unwinding in earnest; enrollment increased in 1.8% in the state over the time period of interest.

In the first year of unwinding, the number of Medicaid redeterminations that ended in termination as a share of pre-unwinding total enrollment varied widely by state (Figure 2). In 6 states, these terminations accounted for less than 15% of total March 2023 enrollment, but in 10 states, terminations exceeded 35% of enrollment at the end of continuous coverage. The median (range) rate was 26.6% (8.3%-55.3%). Net enrollment decreases, accounting for new and returning Medicaid enrollees, were generally smaller, ranging from 1.4% (Maine) to 30.2% (Colorado), with a median of 14.0% (Figure 2). Two states—Oregon, which did not resume procedural terminations until October 2023, and North Carolina, which expanded Medicaid under the Affordable Care Act effective December 1, 2023—saw enrollment gains over this period.^23,24^

**Figure 3.**
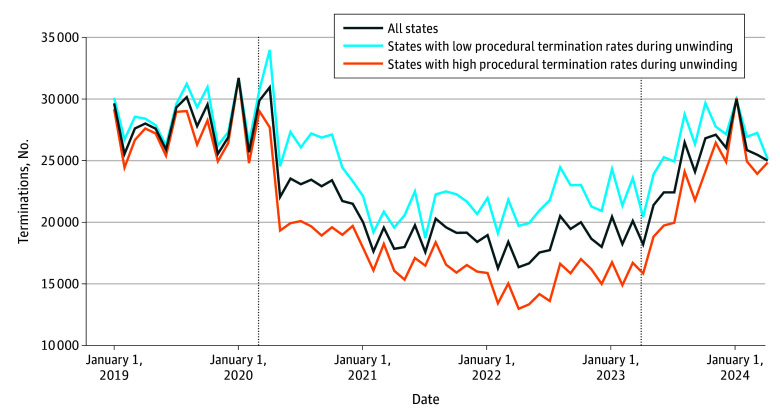
New Medicaid Applications Before, During, and After Continuous Coverage Application data are drawn from monthly US Centers for Medicare & Medicaid Services enrollment reports. States were omitted from analysis if their new applications metric reported to the Centers for Medicare & Medicaid Services included renewals/redeterminations or applications for programs other than Medicaid or if application data were missing (n = 12). States were assigned to the high or low procedural termination category based on whether their procedural termination rate (as a share of completed redeterminations and reported to the Centers for Medicare & Medicaid Services in accordance with the Consolidated Appropriations Act of 2022) was above or below the median for the remaining 41 states (including the District of Columbia). The first dotted line represents May 2020, when the Medicaid continuous coverage provision was implemented, and the second dotted line represents March 2023, when the unwinding began.

The mean number of monthly Medicaid applications was 29.8% lower (from a mean [SD] of 27 914.20 [3669.97] to 19 589.61 [2622.08]) while continuous coverage was in effect (May 2020 to March 2023) relative to the prepandemic period of January 2019 to February 2020 (Table 1). After unwinding began (April 2023 to April 2024), this trend reversed; the mean number of monthly applications was 29.0% higher (from a mean [SD] of 19 589.61 [2622.08] to 25 273.11 [3650.18]) than during continuous coverage (Figure 3; Table 1). When stratifying by state procedural termination rates, the relative increase in applications was larger (32.8%; from a mean [SD] of 20 958.75 [2291.69] to 27 826.14 [3168.97]) in states that had above-median procedural termination rates compared with those that had below-median procedural termination rates (24.7%; from a mean [SD] of 18 220.47 [2200.36] to 22 720.07 [1896.15]). Trends were similar when normalizing each state’s monthly application count to contemporaneous Medicaid enrollment (eFigure and eTable 2 in Supplement 1).

**Table 1.** Mean Number of New Medicaid Applications and Net Change in the Number of Applications by States With Low and High Procedural Termination Rates

Measure	States with low procedural termination rates^a^	States with high procedural termination rates ^a^	All states
New Medicaid applications per mo, mean (SD)^b^			
Prior to continuous coverage (January 2019 to February 2020)	24 894.80 (1751.29)	30 933.60 (2295.70)	27 914.20 (3669.97)
During continuous coverage (May 2020 to March 2023)	18 220.47 (2200.36)	20 958.75 (2291.69)	19 589.61 (2622.08)
During unwinding (April 2023 to April 2024)	22 720.07 (1896.15)	27 826.14 (3168.97)	25 273.11 (3650.18)
Change in applications, %			
After continuous coverage began	−26.81	−32.25	−29.82
After unwinding began	24.70	32.77	29.01

^a^ States were assigned to the high or low procedural termination category based on whether their procedural termination rate (procedural terminations as a share of total terminations) was above or below the median for the remaining 30 states.

^b^ We omitted March and April 2020 due to Medicaid application surges that accompanied the start of the COVID-19 pandemic. States were omitted from the analysis if their new applications metric reported to the US Centers for Medicare & Medicaid Services included extraneous applications, excluded relevant applications, or had missing data (eTable 1 in Supplement 1).

### Variation in Coverage Changes and Cost-Related Barriers to Care

In the interrupted time series model evaluating changes in coverage and access among low-income individuals aged 0 to 64 years using NHIS data (Table 2), the introduction of continuous coverage was not associated with significant increases in self-reported Medicaid overall; among subgroups, only Hispanic respondents reported increased Medicaid enrollment during this period. However, the onset of unwinding was associated with significant reductions in survey-reported Medicaid enrollment (difference per quarter, −1.74 percentage points; 95% CI, −3.43 to −0.06) overall as well as among multiple subgroups, including people in excellent or very good self−reported health (−2.65 percentage points; 95% CI, −4.84 to −0.46), young adults aged 19 to 34 years (−3.37 percentage points; 95% CI, −6.32 to −0.42), White individuals (−2.85 percentage points; 95% CI, −5.36 to −0.34), urban residents (−2.07 percentage points; 95% CI, −3.94 to −0.20), and women (−2.74 percentage points; 95% CI, −4.82 to −0.66). The largest reduction in Medicaid during unwinding was among those currently or recently pregnant (−8.20 percentage points per quarter; 95% CI, −16.35 to −0.03), but the increase in uninsurance among this group was not significant (1.45 percentage points; 95% CI, −3.96 to 6.86).

**Table 2.** Changes in Self-Reported Health Insurance and Cost-Related Barriers to Care Among Low-Income US Residents Aged 0 to 64 Years During the Public Health Emergency (PHE) and Early Unwinding Period, 2019-2023^a^

Characteristic	% (95% CI)					
	Medicaid		Uninsured		Cost barriers	
	PHE trend	Unwinding trend	PHE trend	Unwinding trend	PHE trend	Unwinding trend
Overall	0.44 (−0.38 to 1.25)	−1.74 (−3.43 to −0.06)^b^	−0.92 (−1.56 to −0.28)^c^	1.08 (−0.16 to 2.32)	0.40 (−0.06 to 0.87)	1.06 (0.16 to 1.97)^b^
Sex						
Female	0.92 (−0.09 to 1.94)	−2.74 (−4.82 to −0.66)^b^	−0.91 (−1.72 to −0.10)^b^	0.86 (−0.56 to 2.27)	0.29 (−0.40 to 0.98)	1.20 (−0.09 to 2.49)
Male	−0.09 (−1.27 to 1.09)	−0.55 (−2.93 to 1.83)	−0.92 (−1.85 to 0)	1.33 (−0.51 to 3.18)	0.58 (−0.12 to 1.27)	0.92 (−0.43 to 2.27)
Currently or recently pregnant^d^						
Yes	1.30 (−2.87 to 5.47)	−8.19 (−16.35 to −0.03)^b^	−0.03 (−3.75 to 3.68)	1.45 (−3.96 to 6.86)	0.71 (−2.79 to 4.21)	1.74 (−3.87 to 7.35)
No	0.40 (−0.42 to 1.22)	−1.48 (−3.17 to 0.21)	−0.94 (−1.58 to −0.30)^c^	1.06 (−0.20 to 2.31)	0.40 (−0.07 to 0.86)	1.05 (0.11 to 1.98)^b^
Race and ethnicity^e^						
Hispanic	1.39 (0.12 to 2.67)^b^	−0.68 (−3.49 to 2.14)	−1.82 (−3.08 to −0.56)^c^	0.90 (−1.51 to 3.31)	−0.04 (−0.91 to 0.83)	0.33 (−1.27 to 1.93)
Non-Hispanic American Indian or Alaska Native	0.23 (−6.63 to 7.08)	−3.96 (−16.31 to 8.39)	1.59 (−4.46 to 7.63)	3.63 (−7.05 to 14.31)	−0.55 (−3.16 to 2.07)	0.62 (−5.75 to 6.99)
Non-Hispanic Asian	−1.01 (−4.78 to 2.77)	2.31 (−4.95 to 9.58)	−0.17 (−2.43 to 2.09)	−0.56 (−4.09 to 2.97)	0.97 (−0.65 to 2.59)	−2.61 (−5.38 to 0.15)
Non-Hispanic Black	1.52 (−0.31 to 3.35)	−2.05 (−5.82 to 1.72)	−1.71 (−2.89 to −0.53)^c^	0.61 (−1.85 to 3.06)	0.31 (−0.82 to 1.44)	2.29 (−0.12 to 4.70)
Non-Hispanic White	−0.32 (−1.58 to 0.93)	−2.85 (−5.36 to −0.34)^b^	−0.15 (−1.03 to 0.73)	1.48 (−0.12 to 3.08)	0.47 (−0.27 to 1.20)	1.69 (0.23 to 3.16)^b^
Other race	−0.45 (−4.49 to 3.59)	−3.17 (−11.20 to 4.86)	1.29 (−1.98 to 4.55)	−2.28 (−6.43 to 1.87)	3.25 (1.09 to 5.41)^c^	0.24 (−3.95 to 4.44)
Education						
Less than high school	0.26 (−1.45 to 1.98)	0.19 (−3.21 to 3.59)	−0.55 (−2.23 to 1.14)	−0.06 (−3.17 to 3.06)	0.23 (−1.00 to 1.45)	−0.32 (−2.83 to 2.19)
High school/GED	1.08 (−0.20 to 2.37)	−1.56 (−4.06 to 0.95)	−1.14 (−2.20 to −0.08)^b^	0.20 (−1.61 to 2.01)	0.44 (−0.44 to 1.31)	1.32 (−0.22 to 2.86)
Some college	−0.57 (−1.95 to 0.80)	−2.40 (−5.36 to 0.55)	−0.69 (−1.52 to 0.15)	2.18 (0.13 to 4.24)^b^	0.88 (0.13 to 1.63)^b^	0.98 (−0.70 to 2.66)
College graduate	1.57 (−0.47 to 3.61)	−3.81 (−7.75 to 0.12)	−1.06 (−2.32 to 0.20)	2.54 (−0.17 to 5.24)	−0.43 (−1.41 to 0.55)	2.46 (0.24 to 4.69)^b^
Age, y						
0-18	0.08 (−1.09 to 1.24)	−0.56 (−2.85 to 1.73)	−0.66 (−1.36 to 0.03)	1.15 (−0.22 to 2.52)	0.15 (−0.21 to 0.51)	0.41 (−0.33 to 1.15)
19-34	1.14 (−0.34 to 2.62)	−3.37 (−6.32 to −0.42)^b^	−1.54 (−2.88 to −0.20)^b^	2.32 (−0.24 to 4.88)	0.37 (−0.64 to 1.37)	1.08 (−1.00 to 3.17)
35-44	0.49 (−1.19 to 2.17)	−2.76 (−6.19 to 0.66)	−1.12 (−2.99 to 0.74)	1.71 (−1.65 to 5.06)	0.82 (−0.75 to 2.39)	1.88 (−0.90 to 4.67)
45-54	−0.94 (−2.91 to 1.02)	1.26 (−2.90 to 5.41)	−1.30 (−3.00 to 0.39)	−2.35 (−5.80 to 1.10)	1.60 (−0.09 to 3.30)	0.09 (−3.51 to 3.68)
55-64	1.17 (−0.47 to 2.81)	−2.15 (−5.76 to 1.47)	0.13 (−1.30 to 1.55)	0.38 (−2.25 to 3.00)	−0.18 (−1.61 to 1.26)	2.37 (−0.31 to 5.05)
Geographic						
Rural	−0.71 (−2.60 to 1.18)	−0.14 (−3.88 to 3.61)	−0.85 (−2.56 to 0.87)	0.19 (−2.16 to 2.53)	1.05 (−0.13 to 2.23)	0.50 (−1.75 to 2.76)
Urban	0.68 (−0.22 to 1.57)	−2.07 (−3.94 to −0.20)^b^	−0.93 (−1.62 to −0.24)^c^	1.26 (−0.15 to 2.67)	0.27 (−0.23 to 0.78)	1.18 (0.19 to 2.17)^b^
Self-reported health						
Excellent or very good	0.50 (−0.57 to 1.56)	−2.65 (−4.84 to −0.46)^b^	−1.00 (−1.75 to −0.25)^c^	1.89 (0.22 to 3.55)^b^	0.02 (−0.46 to 0.51)	1.17 (0.16 to 2.19)^b^
Good	−0.17 (−1.65 to 1.31)	−0.44 (−3.34 to 2.46)	−0.85 (−2.07 to 0.38)	0.18 (−2.08 to 2.43)	1.00 (−0.08 to 2.08)	−0.15 (−2.22 to 1.93)
Fair/poor	1.16 (−0.67 to 2.99)	−0.78 (−4.33 to 2.77)	−0.88 (−2.41 to 0.65)	−0.07 (−2.50 to 2.37)	0 (−1.52 to 1.51)	2.79 (−0.03 to 5.61)

Abbreviation: GED, General Educational Development.

^a^ Data are from the National Health Interview Survey, 2019 to 2023 (n = 503 265 663). The sample contains all children and adults aged 0 to 64 years with family incomes at or below 200% of the federal poverty level. The model changes twice in quarterly trends using an interrupted time series; the PHE trend indicates the change from the prepandemic trend starting in the second quarter of 2020 and unwinding trend indicates the change from the pandemic trend starting in the third quarter of 2023. Each time period has its own slope, with a single common intercept. The slope during the time trend (2019 to 2023) serves as the baseline, and the slopes during the PHE trend and during the unwinding trend can be compared with this baseline.

^b^ *P* < .05.

^c^ *P* < .01.

^d^ This variable is based on responses from women only.

^e^ Race and ethnicity are self-reported by survey respondents.

Uninsurance during unwinding did not increase significantly in the full sample of low-income individuals (1.08 percentage points; 95% CI, −0.16 to 2.32) but did rise significantly among healthier individuals (1.89 percentage points; 95% CI, 0.22 to 3.55) and those who attended some college (2.18 percentage points; 95% CI, 0.13 to 4.24). Unwinding was also associated with increased cost-related barriers to care among White individuals (1.69 percentage points; 95% CI, 0.23 to 3.16), college graduates (2.46 percentage points; 95% CI, 0.24 to 4.69), urban residents (1.18 percentage points; 95% CI, 0.19 to 2.17), and healthier individuals (1.17 percentage points; 95% CI, 0.16 to 2.19). We found that women experienced a significant increase in rates of private insurance during the unwinding period (2.11 percentage points; 95% CI, 0.09 to 4.13), although the overall population estimate (including men) for this outcome was not significant (0.82 percentage points; 95% CI, −0.72 to 2.36) (eTable 3 in Supplement 1).

## Discussion

Unprecedented rates of Medicaid disenrollment that began in early 2023 during unwinding have not produced meaningful changes to overall self-reported uninsured rates through the end of 2023. Our findings offer insights about 3 main factors that help explain the disconnect between large Medicaid losses in administrative data and relative stability in overall survey-reported coverage rates, including (1) enrollee confusion, reflected in the worsening survey undercount of Medicaid during the COVID-19 pandemic, which began to correct during unwinding; (2) churning, where people lose Medicaid but reapply and regain coverage; and (3) transitions to alternative coverage. We discuss each of these phenomena in turn.

### Medicaid Undercount and Enrollee Confusion

The growing Medicaid undercount during continuous coverage is suggestive of enrollee confusion, which could partly explain the relative stability of the uninsured rate during unwinding. States and the federal government recorded pandemic-era increases in Medicaid enrollment that increasingly exceeded the number of US residents aware of their own Medicaid enrollment. Prior research suggests that this reflects that some people were reporting incorrectly that they were uninsured when they remained enrolled in Medicaid.^4,11^ Our findings from the NHIS are consistent with recent research examining US Census data and finding a similar pattern during the COVID-19 pandemic.^16^ Our analysis adds evidence that the undercount began to narrow again once unwinding resumed, likely reflecting that at least some of the people having Medicaid benefits terminated had already stopped thinking of themselves as Medicaid enrollees, including people who transitioned to commercial coverage.^25^

Notably, our results find the major increase in the undercount during the COVID-19 pandemic occurred among children 18 years and younger—with an undercount that, at its peak, was roughly 3-fold larger than that for adults. Future research could help characterize parents’ understanding of their children’s coverage over time. This is a particularly timely issue for policymakers to consider since all states are now required to provide 12 months of continuous eligibility to all children in Medicaid, with multiple states contemplating multiyear continuous eligibility policies for children.^26,27,28,29^ Beneficiary confusion could lead to continuous coverage policies failing to produce intended improvements in access to care, as well as states continuing to pay managed care plans for enrollees not using their coverage.

### Medicaid Churn

Terminations during unwinding, calculated as a share of March 2020 enrollment, were on average almost twice the size of net enrollment change over the same time period. This difference reflects, in part, new enrollment among people who had not been enrolled in the program during the PHE—but it also reflects re-entry of people who lost Medicaid during unwinding despite remaining eligible or who experienced very short-term eligibility fluctuations. While we cannot directly measure Medicaid re-entry among people who had their coverage terminated, prior work has found that 1 in 5 adults and more than 1 in 3 children re-enter Medicaid within 6 months of program exit.^30,31^

We found a sharp and sustained increase in new Medicaid applications after unwinding began, consistent with a resumption of churn. The average increase in Medicaid applications during unwinding was higher in states with more procedural determinations as a share of completed redeterminations, suggesting that states that were less able to ascertain eligibility—perhaps because of state policy and unwinding implementation choices—experienced higher rates of churn, with implications for both enrollees and states. Episodes of Medicaid churn produce smaller changes in the uninsured rate than more permanent losses of coverage, but even short coverage gaps have been associated with adverse changes to health care access, and prior research estimated that an episode of churn cost states between $400 and $600 (in 2015 dollars).^32,33,34,35,36,37,38^

### Heterogeneity in Coverage Outcomes

Our interrupted time series analysis found that lower-income individuals aged 0 to 64 years experienced smaller changes in the uninsured rate during unwinding than reductions in self-reported Medicaid. Although the increases in uninsurance and private coverage for this group did not reach statistical significance, the point estimates are illustrative: compared with a 1.74–percentage point reduction (95% CI, −3.43 to −0.06) per quarter in self-reported Medicaid during unwinding, the uninsured rate rose by 1.08 percentage points (95% CI, −0.16 to 2.32) and private insurance rose by 0.82 percentage points (95% CI, −0.72 to 2.36), which is potentially compatible with previous findings that roughly one-half of low-income adults losing Medicaid during unwinding became uninsured.^7^

Meanwhile, among women, gains in private coverage were more pronounced, increasing 2.11 percentage points (95% CI, 0.09 to 4.13), while Medicaid coverage fell 2.74 percentage points (95% CI, −4.82 to −0.66). It is not entirely clear why coverage changes were larger for women. In part, this appears to pertain to pregnancy-related coverage, as pregnant or recently pregnant individuals had the highest rates of Medicaid loss in the survey (−8.20 percentage points [95% CI, −16.35 to −0.03] per quarter) but did not experience a significant increase in the uninsured rate. The continuous coverage provision may have kept many women in Medicaid after pregnancy who would otherwise have transitioned to other coverage rather than becoming uninsured. Again, this raises implications for beneficiary outreach and education, since nearly all states have recently adopted a new option to provide 12 months of continuous Medicaid coverage in the postpartum period.^39,40^

Looking at other subgroups, self-reported Medicaid losses were concentrated among younger adults, those in better self-reported health, urban residents, and non-Hispanic White individuals. To some extent, these patterns—particularly among younger and healthier individuals—may reflect groups less consistently engaged in health care and for whom health insurance may be less salient. Nonetheless, we detected signs of worsening health care affordability during unwinding for these same groups—White, college educated, and healthier individuals. Future research with more granular data on health care utilization and health outcomes is needed to better understand potential harms of the coverage disruptions caused by unwinding. These results also should serve as a note of caution that recent legislative proposals to increase eligibility determinations within Medicaid or otherwise increase administrative barriers for enrolling or maintaining coverage, such as work requirements, may create significant affordability concerns among these populations.^41,42^

States were generally expected to complete unwinding by mid or late 2024. In May 2024, CMS announced that it would require states to continue reporting redetermination outcomes into the future, after unwinding. These metrics, which were not publicly available before unwinding, will help policymakers and researchers alike better understand how the eligibility redetermination process affects continuity of coverage.

The disconnect between perceived and actual enrollment in Medicaid has important policy implications, particularly in the context of most states extending postpartum eligibility, a new 12-month continuous eligibility requirement for children, and certain states extending child eligibility for more than a year.^39,43^ Continuous eligibility policies may not have the intended effect on access to coverage if beneficiaries do not understand that they remain enrolled in the program. Additional qualitative and quantitative research is needed to understand the best ways to communicate with enrollees about benefit continuity. Such research will also be critical if proposals to shorten windows of Medicaid eligibility—by mandating more frequent eligibility redeterminations, for example—are passed into law, potentially resulting in the opposite situation: enrollees believing they still have Medicaid (and perhaps seeking care) when their coverage has in fact lapsed.

### Limitations

Our study has limitations. Some terminations were still unresolved (ie, pending) at time of report, although we minimized this limitation by using reports that were updated 3 months after renewals were due to reflect new outcomes and corrections.^44^ Additionally, starting in February 2024, redetermination outcomes reflected both enrollees who were redetermined as part of unwinding and enrollees who entered Medicaid after continuous coverage had ended and underwent routine annual redeterminations.

Our interrupted time series analysis for individuals aged 0 to 64 years with household incomes below 200% the federal poverty level may not generalize to older adults or individuals with higher incomes who also potentially were affected by the continuous coverage and unwinding policies. NHIS data were only publicly available through 2023; it is possible some people did not experience coverage losses until 2024. Finally, our analytic approach was not designed to support causal inference; findings should be understood as associations.

## Conclusions

In this cross-sectional study, although the unwinding of Medicaid continuous coverage represented a potentially seismic shift in the US health insurance landscape with more than 25 million people being terminated from the program,^6^ self-reported uninsured estimates remained much steadier through the end of 2023. Our findings suggest that enrollee confusion, re-enrollment in Medicaid, and transitions to alternative coverage each contributed to this apparent contradiction, with some notable variation across subgroups.

## Notes

Supplement 1. **eMethods.** Interrupted Time Series Regression Approach **eTable 1.** States Included and Excluded in Application Analyses **eFigure.** New Medicaid Applications as a Share of Monthly Enrollment **eTable 2.** Mean Medicaid Application Rate and Net Change Rate in the Number of Applications by States With Low and High Procedural Termination Rates **eTable 3.** Changes in Self-Reported Health Insurance Among Low-Income US Residents (0-64) During the Public Health Emergency (PHE) and Early Unwinding Period, 2019-2023

Supplement 2. Data Sharing Statement

## References

[1] Nelson DB, Goldman AL, Zhang F, Yu H. Continuous Medicaid coverage during the COVID-19 public health emergency reduced churning, but did not eliminate it. Health Aff Sch. 2023;1(5):qxad055. doi:10.1093/haschl/qxad055 38223316 PMC10786332

[2] Dague L, Ukert B. Pandemic-era changes to Medicaid enrollment and funding: implications for future policy and research. J Policy Anal Manage. Published online October 21, 2023. doi:10.1002/pam.22539

[3] Center on Budget and Policy Priorities. Unwinding Watch: tracking Medicaid coverage as pandemic protections end. Accessed January 3, 2025. https://www.cbpp.org/research/health/unwinding-watch-tracking-medicaid-coverage-as-pandemic-protections-end

[4] McIntyre A, Smith RB, Sommers BD. Survey-reported coverage in 2019-2022 and implications for unwinding Medicaid continuous eligibility. JAMA Health Forum. 2024;5(4):e240430. doi:10.1001/jamahealthforum.2024.0430 38578627 PMC10998158

[5] Tolbert J, Rudowitz R, Drake P. Understanding Medicaid procedural disenrollment rates. Accessed December 19, 2024. https://www.kff.org/policy-watch/understanding-medicaid-procedural-disenrollment-rates/

[6] Tolbert J, Corallo B. An examination of Medicaid renewal outcomes and enrollment changes at the end of the unwinding. Accessed January 3, 2025. https://www.kff.org/medicaid/issue-brief/an-examination-of-medicaid-renewal-outcomes-and-enrollment-changes-at-the-end-of-the-unwinding/

[7] McIntyre A, Sommers BD, Aboulafia G, . Coverage and access changes during Medicaid unwinding. JAMA Health Forum. 2024;5(6):e242193. doi:10.1001/jamahealthforum.2024.2193 38943683 PMC11214671

[8] Bensken WP, Koroukian SM, McGrath BM, Alberti PM, Cottrell EK, Sills MR. Unwinding of continuous Medicaid coverage among patients at community health centers. JAMA Health Forum. 2024;5(1):e234622. doi:10.1001/jamahealthforum.2023.4622 38180766 PMC10770764

[9] Marinacci LX, Mein SA, Engel-Rebitzer E, Figueroa JF, Wadhera RK. Medicaid disenrollment by race and ethnicity in the United States: a national cross-sectional analysis. J Gen Intern Med. 2025;40(2):498-500. doi:10.1007/s11606-024-08886-5 39261340 PMC11803064

[10] Rumalla KC, Nelson DB, McConnell KJ, Zhu JM. Racial and ethnic disparities in Medicaid disenrollment after the end of the COVID-19 public health emergency. JAMA Intern Med. 2024;184(8):987-989. doi:10.1001/jamainternmed.2024.1503 38829672 PMC11148781

[11] Ding D, Sommers BD, Glied SA. Unwinding and the Medicaid undercount: millions enrolled in Medicaid during the pandemic thought they were uninsured. Accessed May 30, 2024. https://www.healthaffairs.org/doi/10.1377/hlthaff.2023.01069 10.1377/hlthaff.2023.01069 38709963

[12] Daw JR, MacCallum-Bridges CL, Kozhimannil KB, Admon LK. Continuous Medicaid eligibility during the COVID-19 pandemic and postpartum coverage, health care, and outcomes. JAMA Health Forum. 2024;5(3):e240004. doi:10.1001/jamahealthforum.2024.0004 38457131 PMC10924249

[13] Levitt L. With or without ACA repeal, ACA and Medicaid cuts are looming. JAMA Health Forum. 2024;5(12):e245140. doi:10.1001/jamahealthforum.2024.5140 39636628

[14] Park E. Medicaid on the chopping block. N Engl J Med. Published online February 26, 2025. doi:10.1056/NEJMp2501855 40009824

[15] KFF. Tracking the Medicaid provisions in the 2025 reconciliation bill. Accessed June 5, 2025. https://www.kff.org/tracking-the-medicaid-provisions-in-the-2025-budget-bill/

[16] Gupta S, Behrer C, Wang V, Banthin JS, Bundorf MK. Resumption of Medicaid eligibility redeterminations: little change in overall insurance coverage. Health Aff (Millwood). 2024;43(11):1518-1527. doi:10.1377/hlthaff.2024.00641 39442020

[17] US Census Bureau. Household Pulse Survey technical documentation. Accessed March 2, 2025. https://www.census.gov/programs-surveys/household-pulse-survey/technical-documentation.html

[18] US Centers for Disease Control and Prevention. National Health Interview Survey: 2023 survey description. Accessed March 2, 2025. https://ftp.cdc.gov/pub/Health_Statistics/NCHS/Dataset_Documentation/NHIS/2023/srvydesc-508.pdf

[19] Medicaid.gov. Medicaid and CHIP enrollment trend snapshot. Accessed January 5, 2025. https://www.medicaid.gov/medicaid/program-information/medicaid-chip-enrollment-data/medicaid-and-chip-enrollment-trend-snapshot/index.html

[20] Medicaid.gov. Monthly Medicaid & CHIP application, eligibility determination, & enrollment reports & data. Accessed January 5, 2025. https://www.medicaid.gov/medicaid/national-medicaid-chip-program-information/medicaid-chip-enrollment-data/monthly-medicaid-chip-application-eligibility-determination-and-enrollment-reports-data/index.html

[21] Medicaid.gov. Data reporting: Medicaid and CHIP renewals: returning to regular operations. Accessed January 5, 2025. https://www.medicaid.gov/resources-for-states/coronavirus-disease-2019-covid-19/unwinding-data-reporting/index.html

[22] Zylla E, Lukanen E. States’ reporting of Medicaid unwinding reinstatement data. Accessed June 5, 2025. https://shvs.org/states-reporting-of-medicaid-unwinding-reinstatement-data/

[23] KFF. State approaches to the unwinding period. Accessed February 8, 2024. https://www.kff.org/other/state-indicator/state-approaches-to-the-unwinding-period/

[24] Robertson GD. North Carolina to launch Medicaid expansion on Dec. 1. *The Associated Press*. September 25, 2023. Accessed January 6, 2025. https://apnews.com/article/north-carolina-medicaid-expansion-governor-dfa2b47ef31381f75d2a6e5c72619491

[25] Volkov E, Eloso J, Bosworth A, Finegold K, Buchmueller TC. Medicaid ‘unwinding:’ much of the reduction in Medicaid-paid prescriptions was offset by increased commercial coverage. Health Aff (Millwood). 2025;44(5):523-530. doi:10.1377/hlthaff.2024.01492 40324132

[26] Hogan C, Volkov E, Peters C, Lew ND, Buchmueller T. New federal 12-month continuous eligibility expansion: over 17 million children could gain new protections from coverage disruptions. Accessed January 6, 2025. https://aspe.hhs.gov/sites/default/files/documents/0366907d43ad429094b60fb707ee9825/aspe-childrens-continuous-eligibility.pdf

[27] Burak EW. CMS approves five more states to adopt Medicaid multi-year continuous coverage for young children as threats to coverage loom. Accessed January 6, 2025. https://ccf.georgetown.edu/2024/11/15/cms-approves-five-more-states-to-adopt-medicaid-multi-year-continuous-coverage-for-young-children-as-threats-to-coverage-loom/

[28] Eliason E, Nelson D, Vasan A. Continuous eligibility policies and CHIP structure affected children’s coverage loss during Medicaid unwinding. Health Aff (Millwood). 2025;44(3):288-295. doi:10.1377/hlthaff.2024.01099 40030112 PMC12136013

[29] Chua KP, Constantin J, Kenney GM, Conti RM, Simon K. Changes in chronic medication dispensing to children and young adults during Medicaid unwinding. Pediatrics. 2025;155(6):e2024070380. doi:10.1542/peds.2024-070380 40314398

[30] Dague L, Myerson R. Loss of Medicaid coverage during the renewal process. JAMA Health Forum. 2024;5(5):e240839. doi:10.1001/jamahealthforum.2024.0839 38700852 PMC11069080

[31] Frenier C, McIntyre A. Insurance coverage transitions after disenrollment from Medicaid in Minnesota. JAMA Netw Open. 2023;6(4):e239379. doi:10.1001/jamanetworkopen.2023.9379 37083669 PMC10122164

[32] Bindman AB, Chattopadhyay A, Auerback GM. Medicaid re-enrollment policies and children’s risk of hospitalizations for ambulatory care sensitive conditions. Med Care. 2008;46(10):1049-1054. doi:10.1097/MLR.0b013e318185ce24 18815526

[33] Bindman AB, Chattopadhyay A, Auerback GM. Interruptions in Medicaid coverage and risk for hospitalization for ambulatory care-sensitive conditions. Ann Intern Med. 2008;149(12):854-860. doi:10.7326/0003-4819-149-12-200812160-00004 19075204

[34] Hall AG, Harman JS, Zhang J. Lapses in Medicaid coverage: impact on cost and utilization among individuals with diabetes enrolled in Medicaid. Med Care. 2008;46(12):1219-1225. doi:10.1097/MLR.0b013e31817d695c 19300311

[35] Abdus S. Part-year coverage and access to care for nonelderly adults. Med Care. 2014;52(8):709-714. doi:10.1097/MLR.0000000000000167 25023915

[36] Sommers BD, Gourevitch R, Maylone B, Blendon RJ, Epstein AM. Insurance churning rates for low-income adults under health reform: lower than expected but still harmful for many. Health Aff (Millwood). 2016;35(10):1816-1824. doi:10.1377/hlthaff.2016.0455 27702954

[37] Gordon SH, Sommers BD, Wilson IB, Trivedi AN. Effects of Medicaid expansion on postpartum coverage and outpatient utilization. Health Aff (Millwood). 2020;39(1):77-84. doi:10.1377/hlthaff.2019.00547 31905073 PMC7926836

[38] Sugar S, Peters C, Lew ND, Sommers BD. Medicaid Churning and Continuity of Care: Evidence and Policy Considerations Before and After the COVID-19 Pandemic. Accessed May 22, 2024. https://aspe.hhs.gov/sites/default/files/private/pdf/265366/medicaid-churning-ib.pdf

[39] Gordon S, Whitman A, Sugar S, . Medicaid after pregnancy: state-level implications of extending postpartum coverage (2023 update). Accessed January 6, 2025. https://aspe.hhs.gov/sites/default/files/documents/168cd047bebc0725da3128104ec8fdde/Postpartum-Coverage-Issue-Brief.pdf 40705906

[40] Varney S. Every state but Arkansas has moved to expand postpartum Medicaid coverage. Accessed January 6, 2025. https://www.npr.org/2024/08/21/nx-s1-5074837/every-state-but-arkansas-has-moved-to-expand-postpartum-medicaid-coverage

[41] Coleman A, Federman S. Work requirements for Medicaid enrollees. Accessed June 23, 2025. https://www.commonwealthfund.org/publications/explainer/2025/jan/work-requirements-for-medicaid-enrollees

[42] Haley JM, Dubay L, Carter J, Zuckerman S. More-frequent Medicaid redeterminations would reduce health insurance coverage and increase administrative costs. Accessed June 23, 2025. https://www.urban.org/urban-wire/more-frequent-medicaid-redeterminations-would-reduce-health-insurance-coverage-and

[43] Buettgens M. Ensuring continuous eligibility for Medicaid and CHIP: coverage and cost impacts for children. Accessed March 4, 2025. https://www.urban.org/research/publication/ensuring-continuous-eligibility-medicaid-and-chip-children

[44] Medicaid.gov. Monthly data reports. Accessed Jun3 5, 2025. https://www.medicaid.gov/resources-for-states/coronavirus-disease-2019-covid-19/unwinding-and-returning-regular-operations-after-covid-19/data-reporting/monthly-data-reports

