# Supplementary material for: US Coverage Changes During Medicaid Unwinding in 2023
Source: JAMA Health Forum. 2025 Oct 10;6(10):e253887. doi: 10.1001/jamahealthforum.2025.3887 (PMC12514626; doi:10.1001/jamahealthforum.2025.3887)
Supplement: Supplement 1. — eMethods. Interrupted Time Series Regression Approach eTable 1. States Included and Excluded in Application Analyses eFigure. New Medicaid Applications as a Share of Monthly Enrollment eTable 2. Mean Medicaid Application Rate and Net Change Rate in the Number of Applications by States With Low and High Procedural Termination Rates eTable 3. Changes in Self-Reported Health Insurance Among Low-Income US Residents (0-64) During the Public Health Emergency (PHE) and Early Unwinding Period, 2019-2023 [file jamahealthforum-e253887-s001.pdf]

Supplemental Online Content

McIntyre A, Morein M, Kim J, Figueroa JF, Sommers BD. US coverage changes during Medicaid unwinding in 2023. *JAMA Health Forum*. 2025;6(10):e253887. doi:10.1001/jamahealthforum.2025.3887

eMethods. Interrupted Time Series Regression Approach
eTable 1. States Included and Excluded in Application Analyses
eFigure. New Medicaid Applications as a Share of Monthly Enrollment
eTable 2. Mean Medicaid Application Rate and Net Change Rate in the Number of Applications by States With Low and High Procedural Termination Rates
eTable 3. Changes in Self-Reported Health Insurance Among Low-Income US Residents (0-64) During the Public Health Emergency (PHE) and Early Unwinding Period, 2019-2023

This supplementary material has been provided by the authors to give readers additional information about their work.

© 2025 McIntyre A et al. *JAMA Health Forum*.

eMethods. Interrupted Time Series Regression Approach

We specified our regression as follows:

$$Y_{it} = \beta_0 + \beta_1 TimeTrendQuarter + \beta_2 PHETrendQuarter + \beta_3 UnwindingTrendQuarter + \epsilon_{it}$$

Y_{it} represents the health insurance coverage such as self-reported Medicaid, uninsurance, and cost-related barriers to care. People were coded as having a cost-related barrier to care if they indicated at least one of the following: (1) they had not received care in the past 12 months because they could not afford it, or (2) they had delayed care in the past 12 months because they could not afford it. *TimeTrendQuarter* represents overall time trend from January 2019 to December 2023, *PHETrendQuarter* begins in April 2020, and *UnwindingTrendQuarter* starts in July 2023.

© 2025 McIntyre A et al. *JAMA Health Forum*.

eTable 1. States Included and Excluded in Application Analyses

**Included states (N = 30)**	
High procedural termination rate (< 20.27%)	AL, AR, CO, GA, ID, LA, MA, MI, MN, MT, ND, NH, TX, WA WV
Low procedural termination rate (> 20.27%)	AZ, DC, DE, HI, IL, IN, KY, MD, ME, MS, NC, NE, NM, SC, VT
**Excluded states (N = 21)**	
1. Application count includes renewals or redeterminations	AK, CT, IA, MD, MO, NY, OH, OK, PA, SD, UT, VA
2. Application count does not include all applications to state Medicaid agencies (including failure to include applications for limited benefit programs)	CA, CT, FL, NJ, OR, SD, TN, WI, WY
4. Application count included other programs	RI, UT
5. Application data missing	KS, NY

Notes: Procedural termination rates were calculated by taking the mean of monthly procedural termination rates as a share of redeterminations due; the median procedural termination rate among included states was 20.27%. Exclusions based on notes for the new application variable in CMS enrollment reports, states were excluded If their application count met exclusion criteria 1–3 in any month. States were excluded if they appeared to have missing application data (populating zero applications) in more than two months over the time horizon of interest (January 2019-April 2024). Only one state had minor missingness (TN, in January and February 2019).

© 2025 McIntyre A et al. *JAMA Health Forum*.

eFigure. New Medicaid Applications as a Share of Monthly Enrollment

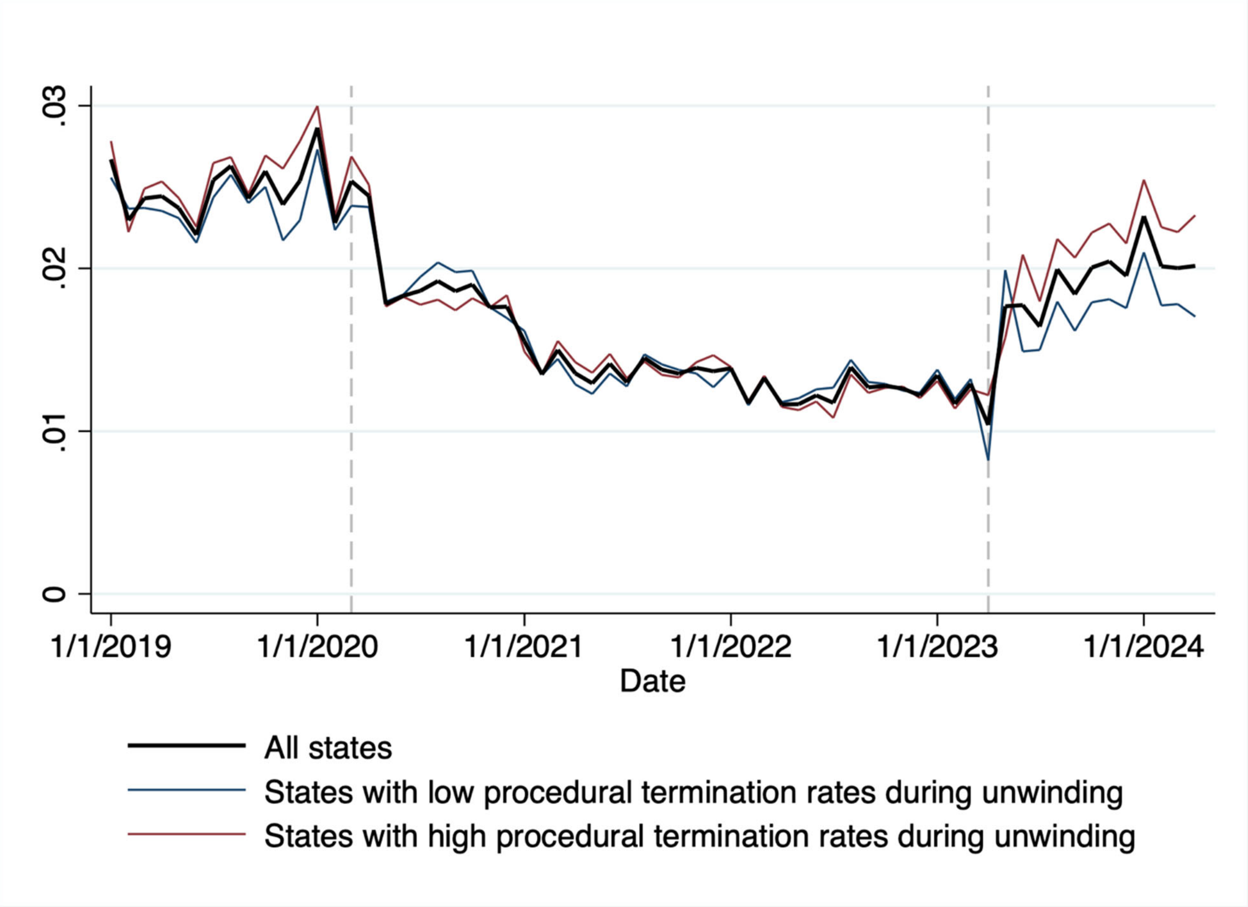

Notes: Application data are drawn from monthly CMS enrollment reports for 30 states that met inclusion criteria. Applications for each state-month are divided by total Medicaid enrollment in that state and month, to account for differences in Medicaid population size. See table S1 for included and excluded states. The first vertical line represents May 2020, when the Medicaid continuous coverage provision was implemented, and the second vertical line represents March 2023, when the unwinding began.

© 2025 McIntyre A et al. *JAMA Health Forum*.

eTable 2. Mean Medicaid Application Rate and Net Change Rate in the Number of Applications by States With Low and High Procedural Termination Rates

	States with low procedural termination rates	States with high procedural termination rates	All states
**Mean Medicaid application rate (applications as a share of monthly enrollment)**			
Prior to unwinding (January 2019-February 2020)	2.39%	2.57%	2.48%
During continuous coverage (May 2020-March 2023)	1.45%	1.42%	1.44%
During unwinding (April 2023-April 2024)	1.76%	2.14%	1.95%
**Changes in the application rate**			
After continuous coverage begins	-39.47%	-44.54%	-42.09%
After unwinding begins	21.55%	50.52%	35.91%

Notes: We omit March and April 2020 due to Medicaid application surges that accompanied the start of the COVID-19 pandemic. States were omitted from the analysis if their "new applications" metric reported to CMS included renewals/redeterminations or other program applications (N =10). States were assigned to the "high" or "low" procedural termination category based on whether their procedural termination rate (as a share of completed redeterminations) was above or below the median for the remaining 41 states (including the District of Columbia).

eTable 3. Changes in Self-Reported Health Insurance Among Low-Income US Residents (0-64) During the Public Health Emergency (PHE) and Early Unwinding Period, 2019-2023

	**Usual Source**		**Private Insurance**	
**Characteristics**	PHE trend % (95% CI)	Unwinding trend % (95% CI)	PHE trend % (95% CI)	Unwinding trend % (95% CI)
Overall	-0.44 (-1.02 to 0.13)	0.68 (-0.51 to 1.87)	0.66 (-0.06 to 1.38)	0.82 (-0.72 to 2.36)
Sex				
Male	-0.91 (-1.78 to -0.04)*	1.53 (-0.27 to 3.33)	1.38 (0.29 to 2.48)*	-0.7 (-2.72 to 1.33)
Female	0 (-0.72 to 0.71)	-0.02 (-1.52 to 1.49)	0 (-0.89 to 0.9)	2.11 (0.09 to 4.13)*
Current / Recently pregnant‡				
Yes	-3.51 (-7.04 to 0.02)	3.31 (-3.74 to 10.35)	-2.04 (-5.44 to 1.36)	5.33 (-1.46 to 12.12)
No	-0.35 (-0.93 to 0.23)	0.58 (-0.62 to 1.79)	0.74 (0.02 to 1.46)*	0.65 (-0.91 to 2.21)
Race/Ethinicity				
Hispanic	-0.63 (-1.68 to 0.43)	1.41 (-0.7 to 3.52)	0.46 (-0.71 to 1.64)	-0.02 (-2.44 to 2.4)
Non-Hispanic White	-0.26 (-1.04 to 0.52)	-0.13 (-1.89 to 1.64)	0.54 (-0.66 to 1.75)	1.86 (-0.78 to 4.49)
Non-Hispanic Black	-0.28 (-1.64 to 1.07)	2.53 (-0.03 to 5.09)	0.62 (-1 to 2.24)	0.24 (-3.18 to 3.67)
Non-Hispanic Asian	0.86 (-1.43 to 3.15)	-7.58 (-13.52 to -1.64)*	0.09 (-3.52 to 3.71)	0.36 (-6.7 to 7.41)
Non-Hispanic AIAN†	-0.64 (-4.24 to 2.95)	-0.46 (-8.85 to 7.93)	1.81 (-1.02 to 4.65)	-2.87 (-10.91 to 5.16)
Other	-3.06 (-5.74 to -0.38)*	4.04 (-0.74 to 8.82)	1.23 (-2.02 to 4.48)	5.49 (-1.9 to 12.89)
Education				
Less than High school	-0.95 (-2.4 to 0.51)	2.03 (-0.7 to 4.77)	-0.03 (-1.34 to 1.28)	0.35 (-2.09 to 2.78)
High school / GED	-0.14 (-1.07 to 0.79)	1.86 (-0.03 to 3.76)	0.46 (-0.67 to 1.6)	1.28 (-1.11 to 3.68)
Some College	-0.73 (-1.62 to 0.15)	0.14 (-1.7 to 1.98)	1.47 (0.05 to 2.88)	-0.19 (-3.04 to 2.67)
College Graduate	-0.24 (-1.5 to 1.02)	-3.09 (-6.03 to -0.16)*	-0.04 (-2.02 to 1.94)	2.5 (-1.55 to 6.55)
Age				
0-18	-0.14 (-0.72 to 0.44)	-0.3 (-1.59 to 0.99)	0.85 (-0.18 to 1.89)	-0.24 (-2.16 to 1.68)
19-34	0.47 (-0.83 to 1.77)	1.34 (-1.25 to 3.92)	0.56 (-0.95 to 2.08)	1.64 (-1.36 to 4.64)
35-44	-2.01 (-3.58 to -0.45)*	2.62 (-0.48 to 5.73)	0.41 (-1.23 to 2.06)	1.1 (-2.27 to 4.46)
45-54	-0.44 (-2.08 to 1.19)	-0.94 (-4.72 to 2.84)	2.4 (0.49 to 4.32)*	1.42 (-2.74 to 5.57)
55-64	-1.35 (-2.68 to -0.03)*	1.64 (-0.79 to 4.06)	-1.07 (-2.55 to 0.41)	0.61 (-2.75 to 3.97)
Geographic				
Rural	0.14 (-1.03 to 1.31)	0.82 (-1 to 2.63)	2.3 (0.81 to 3.79)**	0.42 (-3.06 to 3.9)
Urban	-0.57	0.67	0.31	0.92

© 2025 McIntyre A et al. *JAMA Health Forum*.

	(-1.22 to 0.08)	(-0.71 to 2.05)	(-0.49 to 1.11)	(-0.8 to 2.63)
Self-reported health				
Excellent/Very good	-0.09<br>(-0.76 to 0.58)	0.95<br>(-0.51 to 2.4)	0.8<br>(-0.18 to 1.78)	1.25<br>(-0.83 to 3.32)
Good	-1.1<br>(-2.26 to 0.05)	0.86<br>(-1.44 to 3.15)	0.95<br>(-0.35 to 2.26)	0.35<br>(-2.16 to 2.86)
Fair/Poor	-0.55<br>(-1.75 to 0.66)	-0.67<br>(-3.49 to 2.15)	0.1<br>(-1.17 to 1.37)	0.12<br>(-3 to 3.24)

Notes: Data are from the National Health Interview Survey, 2019-2023 (N= 503,265,663). Sample contains all children and adults ages 0-64 with family incomes at or below 200% of the Federal Poverty Level. The model two changes in quarterly trends using an interrupted time series: *PHE Trend* indicating the change from the pre-pandemic trend starting in the second quarter of 2020; and *Unwinding Trend* indicating the change from the pandemic trend starting in the third quarter of 2023.
†AIAN = American Indian or Alaska Native
‡This variable is based on responses from women only
*** p < 0.001, **p<.01, *p<.05
