# Supplementary material for: US Coverage Changes During Medicaid Unwinding in 2023
Source: JAMA Health Forum. 2025 Oct 10;6(10):e253887. doi: 10.1001/jamahealthforum.2025.3887 (PMC12514626; doi:10.1001/jamahealthforum.2025.3887)
Supplement: Supplement 2. — Data Sharing Statement [file jamahealthforum-e253887-s002.pdf]

Data Sharing Statement

McIntyre. US Coverage Changes During Medicaid Unwinding in 2023. *JAMA Health Forum*. Published October 10, 2025. doi:10.1001/jamahealthforum.2025.3887

Data
Data available: No

Additional Information
Explanation for why data not available: All data are already publicly available.
